# Mitral Tissue Inhibitor of Metalloproteinase 2 Is Associated with Mitral Valve Surgery Outcome

**DOI:** 10.1371/journal.pone.0086287

**Published:** 2014-01-27

**Authors:** Tsung-Hsien Lin, Sheau-Fang Yang, Chaw-Chi Chiu, Ho-Ming Su, Wen-Chol Voon, Chee-Yin Chai, Wen-Ter Lai, Sheng-Hsiung Sheu

**Affiliations:** 1 Division of Cardiology, Department of Internal Medicine, Kaohsiung Medical University, Kaohsiung, Taiwan; 2 Department of Pathology, Kaohsiung Medical University, Kaohsiung, Taiwan; 3 Division of Cardiovascular Surgery, Department of Surgery, Kaohsiung Medical University, Kaohsiung, Taiwan; 4 Kaohsiung Medical University Hospital, Department of Pathology, Kaohsiung Municipal Ta-Tung Hospital, Kaohsiung Medical University, Kaohsiung, Taiwan; 5 Faculty of Medicine, Kaohsiung Medical University, Kaohsiung, Taiwan; University of Portsmouth, School of Pharmacy & Biomedical Sciences, United Kingdom

## Abstract

**Background:**

Matrix metalloproteinases play a role in regulating cardiac remodeling. We previously reported an association between tissue inhibitor of metalloproteinase 2 (TIMP-2) expression and mitral valve (MV) disease. However, the determinants and prognostic value of mitral TIMP2 after MV surgery are unknown.

**Methods:**

This retrospective study of 164 patients after MV surgery in a tertiary medical center in Taiwan assessed mitral TIMP2 on a semiquantitative scale (0–2) by immunohistochemical staining. The primary endpoints were the composite of cardiovascular death and heart failure admission.

**Results:**

Mean age was 50.4±13.7 years. After a mean follow-up period of 101±59 months, primary endpoints had occurred in 25 (15.2%) subjects. Patients with and without primary endpoint events significantly differed in terms of age (56.6±14.4 vs. 49.2±13.4 years, respectively; p = 0.013) and left ventricular end-systolic diameter (LVESD) (39.7±8.2 vs. 35.5±7.5 mm, p = 0.010) at surgery. The TIMP2 had a significant dose-dependent association with development of a primary endpoint (p = 0.002). Kaplan–Meier analysis showed that TIMP2 expression has a significant positive association with primary endpoint-free survival (log-rank test; p = 0.004). Cox regression analysis showed that independent predictors of primary endpoints were TIMP2 (hazard ratio [HR] 0.28; 95% confidence interval [CI] 0.12–0.65; p = 0.003), age (HR 1.05; 95% CI 1.02–1.09; p = 0.003) and LVESD (HR 1.05; 95% CI 1.01–1.10; p = 0.020).

**Conclusions:**

The lack of mitral TIMP2 expression is associated with increases in cardiovascular death and heart failure following MV surgery.

## Introduction

Tissue turnover plays an important role in the operational longevity of heart valves. The clinical and histopathological features of mitral valve (MV) diseases indicate that matrix degradation and remodeling may be important factors in their severity. Matrix metalloproteinases (MMP) and tissue inhibitor of metalloproteinases (TIMP) contribute to tissue remodeling in several physiological and pathological states [1]–[2]. A recent study found that both matrix synthesis and degradation modify the collagen arrangement in the MV and disrupt its structural and physical properties [3].

Mitral valve surgery can repair valve damage but cannot correct the underlying causes of degenerative disease. Thus, progression of the disease and degradation of the mitral structure due to matrix degeneration may cause late complications. Expression of TIMP2 reportedly stimulates fibroblast growth in the MV [4]–[6]. In addition to regulating MMP2 activity, TIMP2 is also known to inhibit other MMPs, such as gelatinase and collagenase [7]–[8]. The TIMP2 also plays a key role in post-MI myocardial remodeling and exacerbates cardiac dysfunction and remodeling after pressure overload [9]–[10].

This study continues our earlier studies of risk factors for MV disease [11]–[12]. Previous cross-sectional investigation established an association between TIMP2 and mitral valve disease but not causality and no outcome data. Because a clear understanding of valvular matrix expression in response to hemodynamic change may reveal new valvular disease managements, this study investigated the potential role of TIMP2 as a surrogate marker associated with cardiovascular events after MV surgery.

## Methods

### Subject recruitment and baseline data collection

This cross-sectional study retrospectively reviewed the medical files of 164 patients who had received MV surgery at Kaohsiung Medical University Hospital, a tertiary medical center in Taiwan, between June 1, 1991 and November 31, 2006. Baseline data collection for each patient included gender, age, disease duration from symptom onset to surgery, and possible underlying predisposing factors for MV disease, including history of infective endocarditis, MV prolapse, rheumatic heart disease (RHD), major MV disease type [i.e., mitral regurgitation (MR) or mitral stenosis], hypertension, atrial fibrillation, and pulmonary edema. The study was approved by the institutional review board of Kaohsiung Medical University Hospital (KMUH-IRB-960288). According to the local law, no informed consent is required to perform this type of analysis after decoding data.

### Diagnosis of MV disease

Mitral valve disease was diagnosed according to standard echocardiographic methods and surgical findings. Left atrial diameter (LAD), left ventricular end-diastolic diameter (LVEDD) and left ventricular end-systolic diameter (LVESD) were determined by transthoracic echocardiography based on criteria established by the American Society of Echocardiography. The echocardiograph readers were blinded to all baseline patient data.

### Immunohistochemistry

**Figure 1:**
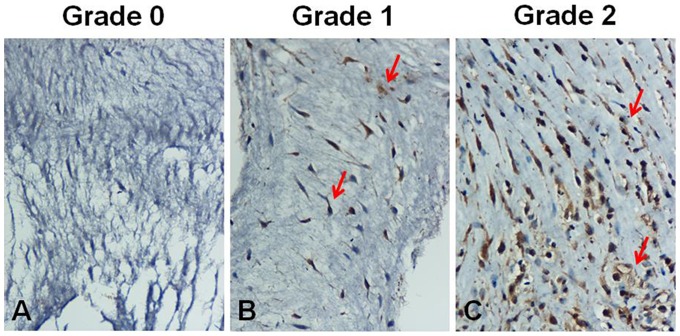
Semiquantitative scoring system for TIMP2 immunostaining. The samples were ranked into 3 grades based on the percentage of positive cells (see arrows): grade 0, negative (A); grade 1, positive staining in small number of cells (B); grade 2, positive staining in large number of cells (C) (original magnification 400×). TIMP2, tissue inhibitor of metalloproteinase.

The results were independently evaluated by an experienced pathologist blinded to all clinical data for the patients. The tissues were obtained from the excised anterior mitral cusp during replacement. Representative tissues were sectioned into blocks (thickness, 4 μm), deparaffinized with xylene, and rehydrated into distilled H_2_O through graded alcohol. Antigen retrieval was enhanced by autoclaving slides in sodium citrate buffer (pH 6.0) for 20 minutes. Endogenous peroxidase activity was quenched by five-minute incubation in 3% hydrogen peroxide. The slides were then incubated with primary human TIMP-2 affinity purified polyclonal antibody (R&D systems, USA) at a dilution of 1∶50 for 1 hour at room temperature [13]. Slides were washed three times in phosphate buffer solution and further incubated with a biotinylated secondary antibody for 60 minutes at room temperature. Antigen-antibody complexes were detected by avidin-biotin-peroxidase method with diaminobenzidine as a chromogenic substrate (DAKO, CA). Finally, the slides were counterstained with hematoxylin and then examined by light microscopy. A negative control was obtained by substituting the primary antibody with the immunoglobulin fraction of non-immune mouse serum in each staining run. Figure 1 shows the staining results, which were assessed on the following semi-quantitative scale: 0 = negative, 1 = positive staining in small number of cells, and 2 =  positive staining in large number of cells.

### Statistical analysis

All data were expressed as mean ± standard deviation. In group comparisons, a chi-square test was used for categorical variables and Student t test was used for continuous variables. The primary outcomes were the composite of cardiovascular death and admission for heart failure. Linear-by-linear association analysis was conducted to find the dose-dependent influence of TIMP2 expression on the occurrence of primary outcomes. Cumulative curves constructed by Kaplan-Meier test were compared by log-rank test. The hazard ratios (HRs) for the primary endpoints were obtained by univariate and multivariate Cox regression analyses. Receiver operating characteristic (ROC) curves were constructed to illustrate the discrimination threshold of continuous variables for the occurrence of primary endpoints. Accuracy is measured by the area under the ROC curve. The Statistical Package for the Social Sciences (SPSS) 11.0 for Windows (SPSS Inc, Chicago, Illinois, USA) was used for all statistical analyses. All tests were 2-sided, and a p value less than 0.05 was considered statistically significant.

## Results

### Baseline characteristics

The analysis included 164 patients who had received MV surgery. After a mean follow-up period of 101±59 months, primary endpoint events occurred in 25 (15.2%) subjects, including 6 cardiovascular death and 19 admission for heart failure.

The baseline characteristics between patients with and without primary endpoint events were shown in Table 1. Compared to patients without primary endpoint events, those with primary endpoint events were significantly older (56.6±14.4 vs. 49.2±13.4 years, respectively; p = 0.013) and had significantly larger LVESD at surgery (39.7±8.2 vs. 35.5±7.5 mm, p = 0.010). Notably, all patients received similar cardiovascular drugs at the time of surgery.

**Table 1:** Comparison of Baseline Characteristics and Medication between Patients with and without Primary Endpoint Events.

Primary endpoints	(−)	(+)	
Parameters	(n = 139)	(n = 25)	p Value
Gender (male, %)	45.3	40.0	0.622
Age (years)	49.2±13.4	56.6±14.4	0.013
Disease duration (months)	44.2±68.1	56.0±96.2	0.458
Systolic blood pressure (mmHg)	120.3±14.7	122.2±15.2	0.553
Diastolic blood pressure (mmHg)	73.9±9.9	76.8±10.4	0.185
LAD (mm)	50.2±10.9	50.3±7.9	0.957
LVEDD (mm)	56.0±9.9	59.0±8.1	0.149
LVESD (mm)	35.5±7.5	39.7±8.2	0.010
Mitral stenosis (%)	37.4	48.0	0.318
Mitral regurgitation (%)	62.6	52.0	
Mitral regurgitation (severity grade)	2.55±1.40	2.48±1.45	0.810
Infective endocarditis (%)	16.5	4.0	0.102
Mitral valve prolapse (%)	7.9	4.0	0.489
Chordae tendinae rupture (%)	36.0	24.0	0.245
Rheumatic heart disease (%)	19.4	24.0	0.599
Coronary artery disease (%)	9.4	12.0	0.681
Hypertension (%)	31.7	40.0	0.414
Smoking (%)	30.9	20.0	0.269
Atrial fibrillation (%)	43.2	52.0	0.413
Fever (%)	13.7	4.0	0.174
Dyspnea (%)	83.5	92.0	0.274
Pulmonary edema (%)	12.2	8.0	0.543
Medication			
RAS blocker (%)	22.3	24.0	0.852
ACEI (%)	16.5	20.0	0.673
ARB (%)	5.8	8.0	0.666
CCB (%)	8.6	8.0	0.917
β-blocker (%)	11.5	0	0.135
Diuretic (%)	43.9	52.0	0.453

LAD, left atrial diameter; LVESD, left ventricular end-systolic dimension; LVEDD, left ventricularend-diastolic dimension; RAS, renin-angiotensin system; ACEI, angiotensin-converting enzyme inhibitors; ARB, angiotensin receptor blockers; CCB, calcium channel blocker.

### Factors associated with TIMP2 expression

Comparisons of mitral TIMP2 expression revealed expression grades 0, 1, and 2 in 14, 107 and 43 patients, respectively. Mitral TIMP2 staining was associated with LAD, LVEDD, severity of MR, and history of the following: RHD, infective endocarditis, chordae tendinae rupture, atrial fibrillation, fever, major mitral disease, LAD, and LVEDD. The TIMP2 staining grade correlated negatively with occurrence of primary endpoints (p = 0.001) (Table 2). Expression of TIMP2 was also linearly related to the development of primary endpoints (p = 0.001 by linear-by-linear association analysis).

**Table 2:** Correlation between TIMP2 and primary endpoints.

Primary Endpoints	(−) (n = 139)	(+) (n = 25)	p Value
TIMP2 Grade	1.23±0.55	0.84±0.55	0.001
0	5.8%	24.0%	0.002
1	64.7%	68.0%	
2	29.5%	8.0%	

Mean ± SD; TIMP2, tissue inhibitor of metalloproteinase.

### Cardiovascular outcomes

**Figure 2:**
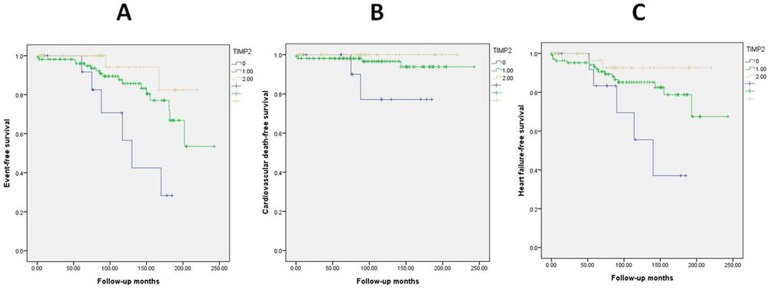
Kaplan-Meier estimates of (A) primary endpoints (B) cardiovascular death (C) admission for heart failure between different TIMP2 expression populations. (p = 0.004, 0.042 and 0.021, respectively). TIMP2, tissue inhibitor of metalloproteinase.

The Kaplan-Meier curves were constructed to compare the three grades of mitral TIMP2 expression in terms of primary endpoint events, cardiovascular death and admission for HF (log rank test p = 0.004, 0.042 and 0.021, respectively) (Figure 2).

**Figure 3:**
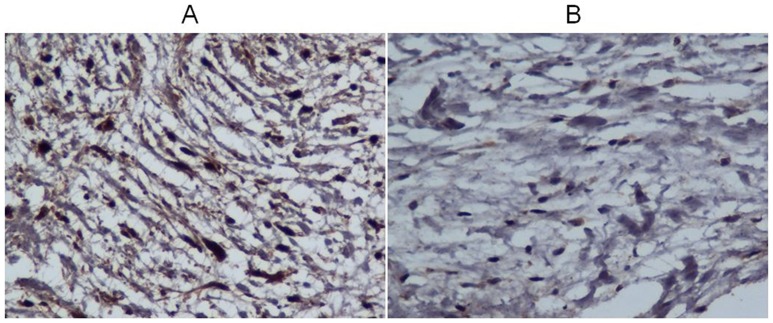
Representative images of TIMP2 expression from patients without and with primary endpoints. (A). Grade 2 (B). Grade 0 (original magnification 400×).

**Figure 4:**
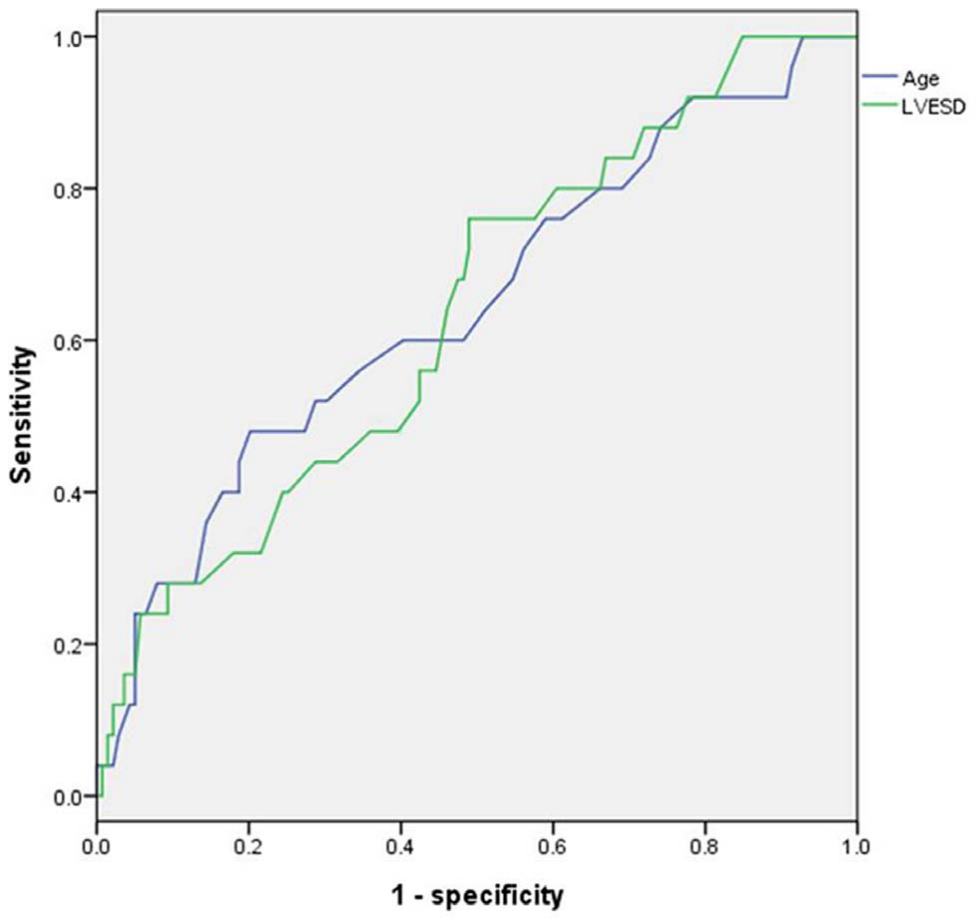
Receiver operating characteristic curves for age and LVESD in the prediction of primary endpoints. LVESD, left ventricular end-systolic diameter.

Cox regression analysis was shown in Table 3. During follow up, the incidence of primary endpoints had a significant negative association with TIMP2 expression (hazard ratio (HR): 0.28; 95% confidence interval (CI): 0.12–0.65; p = 0.003) (Figure 3). Age and LVESD were also independent predictors of primary outcome events (HR: 1.05; 95% CI: 1.02–1.09; p = 0.003 and HR: 1.05; 95% CI: 1.01–1.10; p = 0.020, respectively). ROC curve for predictions of primary endpoints based on age and LVESD was shown in Figure 4. For predicting primary endpoints, the area under the curve values for age and LVESD were 0.644 and 0.636, respectively (p = 0.022 and 0.030, respectively).

**Table 3:** Multivariate Cox regression analysis of independent predictors of primary endpoints.

Factors	Hazard ratio (95% CI)	P value
TIMP2	0.28 (0.12–0.65)	0.003
Age	1.05 (1.02–1.09)	0.003
LVESD	1.05 (1.01–1.10)	0.020

TIMP2, tissue inhibitor of metalloproteinase; LVESD, left ventricular end-systolic dimension.

## Discussion

This study had three major findings. First, mitral TIMP2 staining intensity was associated with the occurrence of primary endpoints, death and HF admission after MV surgery. Second, mitral TIMP 2 staining had a grade-dependent effect on the occurrence of primary endpoints. Third, there was a significant association of TIMP2 expression with the occurrence of primary endpoints even after adjusting the co-variables, including age and LVESD, both of which were also independent predictors of the primary endpoints in this study.

Valvular tissue degeneration is characterized by fibrosis and calcification, which can cause valve dysfunction. The TIMP2 can trigger the signal cascade that instigates cardiac fibrosis, which is a characteristic of MV degeneration. The TIMP2 is also believed to act through specific, high-affinity receptors and through links to G protein and cAMP signaling pathways [14]. Reduction and alkylation of TIMP2 produces a mitogenic and inactive mutant with an additional N-terminal alanine residue related to fibroblast growth [15]–[16]. Lack of TIMP2 exacerbates cardiac dysfunction and impairs remodeling after pressure overload when excess membrane-type MMP activity and loss of integrin β1D degrade the uniformity of extracellular matrix (ECM) remodeling and impair the myocyte–ECM interaction [10]. The pathological findings in our patients showed more cells in the grade 2 TIMP2 section. The higher grade staining with more cells could be associated with TIMP 2 function and might play an important role in the myocardial remodeling. Our study found that lack of mitral TIMP-2 staining is associated with admission for HF and death after MV surgery. These findings suggest that TIMP2 is a prognostic indicator in patients who undergo surgical treatment for MV heart disease.

Animal models have also shown the direct causal roles of TIMP2 activity in left ventricular (LV) remodeling. Heymans et al. showed that mRNA and protein levels of TIMP2 correlate with intra-cardiac fibrosis development [17]. The MMP-inhibitory function of TIMP2 is also a key determinant of myocardial remodeling after MI, mainly due to its inhibition of MT1-MMP.

Replenishing TIMP2 in diseased myocardium has shown potential as a therapeutic treatment for reducing or preventing disease progression [9]. Our data showing that mitral TIMP 2 staining had a grade-dependent effect on the development of primary endpoints supports the continued use of TIMP2 supplement therapy.

Age-dependent changes in LV structure and function may partially result from alterations in TIMP2 expression. Whereas this study showed that age is an independent risk factor for the development of primary endpoints, a previous study found thatTIMP-2 level changes as age increases [18]. These age-dependent alterations in the TIMP-2 profile favor extracellular matrix accumulation and are associated with concentric remodeling and decreased ventricular dysfunction. This association may explain the age-associated increase in the incidence of the primary endpoints in our study.

Another independent predictor of the primary endpoints in this study was LVESD. Previous animal studies have found that, as the LV ejection fraction improves, ventricular remodeling is associated with reduced LVESD and reduced TIMP2 expression, which is consistent with our findings [19]. Compared to the ejection fraction, LVESD (or LV volume) may be less load-dependent and may provide a useful guide for timing MV surgery [20]. Reports of a correlation between preoperative end-systolic diameter and prognosis after MV surgery are also consistent with our data indicating a correlation between LVESD and the occurrence of primary endpoints [21]–[22].

### Limitations

Some limitations of this study are noted. First, this retrospective analysis of a single-center sample was subject to selection bias. Second, TIMP2 expression in tissues was not examined simultaneously with fibrosis-related parameters. Therefore, this study did not determine whether TIMP2 expression is simply a reactive response or a contributing factor in ventricular remodeling. However, this longitudinal study found that TIMP2 has potential use as a prognostic parameter. Third, this study only measured mitral expressions of matrix proteinases. Ventricular expression of proteinases may differ pathologically. However, since the mitral valve and ventricle have the same embryonic origin, they may have similar pathological characteristics. Fourth, proteinase expression was measured only by immunohistochemical analysis. The findings of this require further confirmation by additional measurements such as polymerase chain reaction. Fifth, the findings should be limited to the association of a biomarker with the clinical outcomes because there is no mechanism insight from the current findings. Therefore, the preliminary findings should be replicated if possible.

### Conclusion

Mitral TIMP2 staining proved to be an effective grade-dependent prognostic indicator associated with HF admission and death from MV surgery. These findings suggest that TIMP2 is a potential target for therapeutic intervention in MV disorder after MV surgery.
